# Investigating the footprint of post-domestication dispersal on the diversity of modern European, African and Asian goats

**DOI:** 10.1186/s12711-024-00923-5

**Published:** 2024-07-27

**Authors:** Elena Petretto, Maria Luisa Dettori, María Gracia Luigi-Sierra, Antonia Noce, Michele Pazzola, Giuseppe Massimo Vacca, Antonio Molina, Amparo Martínez, Félix Goyache, Sean Carolan, The AdaptMap Consortium, James Kijas, Bernt Guldbrandtsen, Juha Kantanen, Dylan Duby, Pierre Martin, Coralie Danchin, Delphine Duclos, Daniel Allain, Rémy Arquet, Nathalie Mandonnet, Michel Naves, Isabelle Palhière, Rachel Rupp, François Pompanon, Hamid R. Rezaei, Maeve Foran, Alessandra Stella, Paolo Ajmone-Marsan, Licia Colli, Alessandra Crisà, Donata Marletta, Paola Crepaldi, Michele Ottino, Ettore Randi, Badr Benjelloun, Hans Lenstra, Muhammad Moaeen-ud-Din, Jim Reecy, Isabel Alvarez, Armand Sànchez, Juan Capote, Jordi Jordana, Agueda Pons, Benjamin Rosen, Carina Visser, Cord Drögemüller, Gordon Luikart, Clet Wandui Masiga, Denis Fidalis Mujibi, Hassan Ally Mruttu, Timothy Gondwe, Joseph Sikosana, Maria Da Gloria Taela, Oyekan Nash, Marcel Amills

**Affiliations:** 1 Department of Animal Genetics, Centre for Research in Agricultural Genomics (CRAG), CSIC-IRTA-UAB-UB, Campus Universitat Autònoma de Barcelona, 08193 Bellaterra, Spain; 2 Department of Veterinary Medicine, University of Sassari, 07100 Sassari, Italy; 3 Departament de Ciència Animal i dels Aliments, Universitat Autònoma de Barcelona, 08193 Bellaterra, Spain; 4 Department of Genetics, University of Cordoba, 14071 Córdoba, Spain; 5 Área de Genética y Reproducción Animal, SERIDA-Deva, Camino de Rioseco 1225, 33394 Gijón, Spain; 6 Old Irish Goat Society, Mulranny, Ireland

## Abstract

**Background:**

Goats were domesticated in the Fertile Crescent about 10,000 years before present (YBP) and subsequently spread across Eurasia and Africa. This dispersal is expected to generate a gradient of declining genetic diversity with increasing distance from the areas of early livestock management. Previous studies have reported the existence of such genetic cline in European goat populations, but they were based on a limited number of microsatellite markers. Here, we have analyzed data generated by the AdaptMap project and other studies. More specifically, we have used the geographic coordinates and estimates of the observed (H_o_) and expected (H_e_) heterozygosities of 1077 European, 1187 African and 617 Asian goats belonging to 38, 43 and 22 different breeds, respectively, to find out whether genetic diversity and distance to Ganj Dareh, a Neolithic settlement in western Iran for which evidence of an early management of domestic goats has been obtained, are significantly correlated.

**Results:**

Principal component and ADMIXTURE analyses revealed an incomplete regional differentiation of European breeds, but two genetic clusters representing Northern Europe and the British-Irish Isles were remarkably differentiated from the remaining European populations. In African breeds, we observed five main clusters: (1) North Africa, (2) West Africa, (3) East Africa, (4) South Africa, and (5) Madagascar. Regarding Asian breeds, three well differentiated West Asian, South Asian and East Asian groups were observed. For European and Asian goats, no strong evidence of significant correlations between H_o_ and H_e_ and distance to Ganj Dareh was found. In contrast, in African breeds we detected a significant gradient of diversity, which decreased with distance to Ganj Dareh.

**Conclusions:**

The detection of a genetic cline associated with distance to the Ganj Dareh in African but not in European or Asian goat breeds might reflect differences in the post-domestication dispersal process and subsequent migratory movements associated with the management of caprine populations from these three continents.

**Supplementary Information:**

The online version contains supplementary material available at 10.1186/s12711-024-00923-5.

## Background

Goats were domesticated 10,000 years before present (YBP) in the Fertile Crescent from distinct bezoar populations, a process that was dispersed in time and space but featured by connected human communities [1, 2]. Neolithic goats showed considerable genetic structure associated with geography, so different gene pools were established when human populations with their livestock migrated to Europe, Asia, and Africa [2]. The potential routes of the post-domestication spread of livestock across Europe [3], Asia [4, 5] and Africa [6] have been reported by several authors. Such a dispersal process may cause genetic clines characterized by a decrease in genetic diversity of livestock populations over geographical distance to the domestication area. In European goats, a gradual reduction of genetic diversity with increasing distance to the Fertile Crescent was observed [7, 8], but a limited number of microsatellite markers were used to investigate the patterns of genetic variation in these studies. Here, we have used Illumina Goat SNP50 BeadChip [9] data generated in the AdaptMap project [10] and other studies [11–15] to assess the existence of genetic clines associated with the post-domestication dispersal of goats in Europe, Africa and Asia.

## Methods

### Genotype data

We have used published Illumina Goat SNP50 BeadChip data of European, African and Asian goats generated in the Adaptmap project [10, 16]. In addition to the Adaptmap data, we have also retrieved 50 K data from 473 Swiss goats from 10 different breeds [17]. Moreover, the Old Irish Goat Society based on Mulranny (https://oldirishgoat.ie) provided 50 K data from 383 Old Irish and Old English goats. With regard to African breeds, we retrieved previously published 50 K data from Algerian (N = 48; [11]), Sudanese (N = 72; [12]), and South African (commercial and local breeds N = 114; [13]) goats. Regarding Asian breeds, we combined 50 K data from Chinese (N = 193; [14]) and Iranian (N = 235; [15]) goats. We excluded from our study crossbred populations, and we maintained the number of animals per breed in a range between 15 to 50 individuals (with the only exception of the Carpathian goat, N = 14) by using the “bite.representative.sampling” function of the BITE R package v.2 [18]. This tool preserves the variance structure of the original data set, despite reducing the sample size to a user-defined number. In total, our final data set contained genotype data from 1077 European, 1187 African and 617 Asian goats belonging to 38, 43 and 22 populations, respectively. Observed and expected heterozygosity measurements and geographic coordinates of all goat populations included in the current work are described in Tables 1, 2, 3 and Additional file 1: Figure S1. By using the PLINK v 1.9 software [19] and taking as a reference the goat ARS1 genome [20], the chromosome number, genomic position and name of each SNP were updated, resulting in the retention of 49,376 single nucleotide polymorphisms (SNPs) for European goats, 49,056 SNPs for African goats and 48,898 SNPs for Asian goats. The PLINK v 1.9 software [19] was also used to merge different data sets and filter out uninformative markers *i.e.* (1) SNPs with minor allele frequencies (MAF) lower than 0.05, (2) SNPs with missing call rates higher than 0.05, (3) SNPs that did not fulfil the Hardy–Weinberg expectation (*P* ≤ 0.001), and (4) unmapped SNPs. Moreover, individuals with missing call rates higher than 0.1 were also excluded. After these filtering steps, the African, European and Asian data sets comprised 25,990, 18,135 and 26,888 SNPs respectively. The final total data set (combined data sets of African, European, and Asian breeds) contained, after filtering, 39,030 SNPs genotyped in 2881 goats from 81 breeds.

**Table 1:** Observed and expected heterozygosities, F_ST_, F_is_ and geographic coordinates of the European goat breeds (ordered by country of origin) analyzed in the current work (distances between sampling locations and Ganj Dareh are indicated in km)

ID_Breed	Breed	Country	Long	Lat	N	H_o_	H_e_	F_ST_	F_is_	Distance
LNR_DK	Landrace Goat	Denmark	11.44	55.56	50	0.376	0.391	0.077	0.03887	3631.118
LNR_FI	Landrace Goat	Finland	22.56	62.77	20	0.382	0.384	0.094	0.00353	3613.356
LNR_NL	Landrace Goat	Netherlands	5.12	52.09	15	0.376	0.364	0.131	− 0.03490	3894.742
ENG	Old English Goat	United Kingdom	− 3.44	55.38	32	0.286	0.317	0.126	0.20061	4521.19
CRS	Corse	France	9.00	42.19	29	0.407	0.409	0.056	0.00248	3449.975
FSS	Fosses	France	− 1.12	47.97	24	0.398	0.400	0.068	0.00561	4273.52
PTV	Poitevine	France	0.35	46.50	27	0.387	0.387	0.085	− 0.00197	4155.932
PVC	Provençale	France	4.01	46.28	17	0.418	0.407	0.065	− 0.02903	3873.527
PYR	Pyrenean	France	0.52	43.33	26	0.387	0.395	0.075	0.02034	4143.577
IRL	Old Irish Goat	Ireland	− 8.24	53.41	50	0.346	0.374	0.078	0.06665	4807.525
ARG	Argentata	Italy	15.13	38.02	24	0.423	0.419	0.045	− 0.01119	2925.661
ASP	Aspromontana	Italy	15.91	37.99	23	0.409	0.413	0.051	0.01052	2857.277
BIO	Bionda dell'Adamello	Italy	10.36	46.03	24	0.411	0.411	0.052	− 0.00112	3383.35
CCG	Ciociara Grigia	Italy	13.82	41.61	16	0.406	0.417	0.049	0.02655	3047.132
DIT	Di Teramo	Italy	13.37	42.38	19	0.405	0.383	0.092	− 0.05974	3091.671
GAR	Garganica	Italy	15.57	40.70	15	0.424	0.395	0.075	− 0.07653	2893.263
GGT	Girgentana	Italy	14.17	37.61	24	0.381	0.381	0.091	− 0.00217	3012.074
MLT	Maltese	Italy	14.36	37.60	16	0.380	0.391	0.080	0.02903	2995.381
NIC	Nicastrese	Italy	16.45	38.93	20	0.407	0.417	0.048	0.02321	2809.654
ORO	Orobica	Italy	9.50	46.04	22	0.379	0.375	0.095	− 0.01123	3449.277
RME	Rossa Mediterranea	Italy	15.57	40.70	30	0.425	0.406	0.059	− 0.04794	2893.263
SAR	Sarda	Italy	9.22	39.71	27	0.409	0.413	0.054	0.01004	3432.068
VAL	Valdostana	Italy	7.38	45.71	24	0.382	0.391	0.078	0.02194	3607.712
VSS	Valpassiria	Italy	11.21	46.80	24	0.408	0.413	0.052	0.01119	3334.609
CRP	Carpathian goat	Romania	25.78	46.12	14	0.431	0.428	0.036	− 0.00796	2255.681
BEY	Bermeya	Spain	− 5.26	43.34	23	0.412	0.405	0.067	− 0.01901	4611.009
MAL	Mallorquina	Spain	3.03	39.55	18	0.378	0.391	0.087	0.03235	3963.348
MLG	Malagueña	Spain	− 4.42	37.07	40	0.423	0.417	0.053	− 0.01500	4650.129
RAS	Blanca de Rasquera	Spain	0.61	41.00	20	0.390	0.397	0.078	0.01717	4152.83
ALP_CH	Alpine	Switzerland	7.67	46.95	50	0.410	0.401	0.063	− 0.02508	3603.257
APP	Appenzell	Switzerland	8.23	46.82	29	0.379	0.369	0.104	− 0.03135	3559.048
CHA	Swiss Chamois	Switzerland	8.23	46.82	50	0.413	0.399	0.065	− 0.03628	3559.048
GST	Grisons Striped	Switzerland	8.23	46.82	30	0.405	0.392	0.073	− 0.03355	3559.048
NVE	Nera Verzasca	Switzerland	8.23	46.82	30	0.379	0.375	0.071	− 0.00903	3559.048
PEA	Peacock	Switzerland	8.23	46.82	31	0.408	0.393	0.072	− 0.04171	3559.048
SAA	Saanen	Switzerland	8.23	46.82	30	0.398	0.384	0.087	− 0.02897	3559.048
SGB	Booted goat	Switzerland	8.23	46.82	23	0.395	0.383	0.086	− 0.03326	3559.048
TGR	Tessin grey goat	Switzerland	8.23	46.82	30	0.406	0.399	0.064	− 0.01878	3559.048
TOG	Toggenburg	Switzerland	8.23	46.82	31	0.380	0.372	0.101	− 0.02577	3559.048
VAG	Valais	Switzerland	8.23	46.82	30	0.378	0.373	0.096	− 0.01732	3559.048

Distance, distance (in kilometres) between Ganj Dareh and European sampling locations calculated with the Vincenty ellipsoid-model method (Ganj Dareh: lon 34.27º, lat 47.47º)

Lon: longitude in degrees; Lat: latitude in degrees; N: sample size of each breed; H_o_: Observed heterozygosity; H_e_: Expected heterozygosity; F_ST_: coefficient of genetic differentiation related to the Iranian Markhoz breed; F_is_: inbreeding coefficient

**Table 2:** Observed and expected heterozygosities, F_ST_, F_is_ and geographic coordinates of the African goat breeds (ordered by country of origin) analyzed in the current work (distances between sampling locations and Ganj Dareh are indicated in km)

ID_Breed	Breed	Country	Long	Lat	N	H_o_	H_e_	F_ST_	F_is_	Distance
ALG	Arabia.Makatia.M'Zabite.Kabyle	Algeria	1.67	28.03	48	0.417	0.432	0.037	0.03302	4385.501
SAH	Sahel	Burkina Faso	− 0.39	14.73	15	0.400	0.397	0.058	− 0.00816	5253.199
BUR	Burundi goat	Burundi	29.83	− 2.91	50	0.383	0.382	0.065	− 0.00482	4511.944
CAM	Cameroon Goat	Cameroon	14.39	10.11	37	0.390	0.391	0.057	0.00230	4300.223
WAD_CM	West African Dwarf	Cameroon	10.27	5.9	31	0.371	0.374	0.081	0.00814	4950.331
PAL	Palmera	Canary Islands	− 17.69	28.66	15	0.353	0.348	0.145	− 0.01548	6119.597
BRK	Barki	Egypt	26.9	29.89	50	0.419	0.420	0.030	0.00109	1998.527
OSS	Oasis	Egypt	29.2	26.17	50	0.389	0.411	0.038	0.05391	1971.097
SID	Saidi	Egypt	31.58	26.24	50	0.407	0.417	0.031	0.02514	1766.766
ABR	Abergelle	Ethiopia	38.83	13.33	49	0.399	0.396	0.051	− 0.00903	2478.499
GUM	Gumez	Ethiopia	36.2	12.97	39	0.404	0.400	0.047	− 0.01176	2620.351
KEF	Keffa	Ethiopia	37	7.42	44	0.386	0.392	0.056	0.01433	3161.899
WYG	Woyito Guji	Ethiopia	37.48	5.25	39	0.402	0.399	0.047	− 0.00926	3374.868
GAL	Galla	Kenya	37.66	2.01	23	0.409	0.402	0.044	− 0.01562	3714.419
SEA	Small East African	Kenya	36.97	0.61	30	0.400	0.400	0.044	0.00102	3883.871
MEN	Malagasy goat (Menabe)	Madagascar	45.13	− 20.16	19	0.315	0.316	0.145	0.00021	6029.052
SOF	Malagasy goat (Sofia)	Madagascar	47.67	− 16.74	22	0.321	0.337	0.143	0.04865	5645.415
DZD	Dedza	Malawi	34.33	− 14.37	15	0.351	0.374	0.084	0.06324	5560.364
GUE	Guera	Mali	− 9.19	14.54	16	0.405	0.386	0.078	− 0.05274	6064.181
PEU	Peulh	Mali	− 4.2	14.5	22	0.402	0.392	0.062	− 0.02748	5611.567
SDN	Soudanaise	Mali	− 6.27	13.45	22	0.397	0.392	0.062	− 0.01352	5862.489
TAR	Targui	Mali	− 0.05	16.27	19	0.399	0.396	0.058	− 0.00534	5130.451
MOR	Barcha.Draa.Ghazalia.Morrocan.Noire de l'Atlas.Nord	Morocco	− 7.17	31.09	30	0.405	0.420	0.039	0.03584	5075.599
LND	Landin	Mozambique	32.36	− 25.5	29	0.333	0.344	0.098	0.03190	6805.362
RSK	Red Sokoto	Nigeria	8.17	11.89	19	0.387	0.397	0.054	0.02681	4683.964
SHL	Sahel	Nigeria	8.73	11.25	19	0.402	0.401	0.047	− 0.00009	4680.987
WAD	West African Dwarf	Nigeria	3.74	7.59	19	0.376	0.379	0.082	0.00963	5361.197
SAFR	South Africa Local breeds (from Limpopo.Freestate.Gauteng.Northwest)	South Africa	26.22	− 29.12	26	0.394	0.417	0.038	0.00873	7364.339
DESE	Desert	Sudan	30.37	13.7	24	0.415	0.412	0.038	0.00098	2857.733
NI	Nilotic	Sudan	32.67	13.17	24	0.404	0.410	0.038	− 0.01137	2774.787
TAGG	Taggar	Sudan	29.65	12.05	24	0.407	0.404	0.045	0.04142	3052.918
MAA	Maasai	Tanzania	36.65	− 11.38	18	0.402	0.398	0.050	− 0.00715	5180.503
PRW	Pare White	Tanzania	37.92	− 4.25	19	0.393	0.391	0.061	0.01569	4380.301
SNJ	Sonjo	Tanzania	36.32	− 2.7	20	0.408	0.392	0.058	− 0.00881	4256.064
TUN	Tunisian	Tunisia	9.14	35.74	21	0.419	0.420	0.037	− 0.00994	3480.817
KAR	Karamonja	Uganda	34.67	2.53	19	0.406	0.403	0.044	− 0.00537	3757.129
MUB	Mubende	Uganda	32.29	0.44	18	0.390	0.393	0.059	− 0.04145	4065.861
SEB	Sebei	Uganda	34.45	1.4	21	0.405	0.397	0.052	0.00176	3883.193
MSH	Mashona	Zimbabwe	31.1	− 18.5	22	0.356	0.365	0.078	− 0.00709	6092.687
MTB	Matebele	Zimbabwe	28.51	− 20.55	22	0.410	0.400	0.051	0.00737	6391.261
BOE	Boer	South Africa	28.19	− 25.75	26	0.397	0.405	–	− 0.02110	–
SAV	Savanna	South Africa	23.63	− 29.07	27	0.412	0.411	–	0.02331	–
KHAR	Kalahari Red	South Africa	20.15	− 25.26	22	0.401	0.406	–	− 0.02565	–

Distance, distance (in kilometres) between Ganj Dareh and African sampling locations calculated with the Vincenty ellipsoid-model method (Ganj Dareh: lon 34.27º, lat 47.47º)

Lon: longitude in degrees; Lat: latitude in degrees; N: sample size of each breed; H_o_: Observed heterozygosity; H_e_: Expected heterozygosity; F_ST_: coefficient of genetic differentiation related to the Iranian Markhoz breed; F_is_: inbreeding coefficient

**Table 3:** Observed and expected heterozygosities, F_ST_, F_is_ and geographic coordinates of the Asian goat breeds (ordered by country of origin) analyzed in the current work (distances between sampling locations and Ganj Dareh are indicated in km)

ID_Breed	Breed	Country	Continent	Long	Lat	N	H_o_	H_e_	F_ST_	F_is_	Distance
ANK	Ankara	Turkey	West Asia	31.96	39.97	18	0.415	0.411	0.043	− 0.00878	1514.1681
KIL	Kil	Turkey	West Asia	36.62	40.47	23	0.422	0.416	0.037	− 0.01705	1180.8027
KLS	Kilis	Turkey	West Asia	37.12	36.72	36	0.417	0.415	0.033	− 0.00422	977.4969
IRA_KUR	Markhoz	Iran	West Asia	46.98	35.32	50	0.410	0.404	–	0.05374	124.337
BAB	Barbari	Pakistan	West Asia	72.48	30.30	16	0.387	0.363	0.116	− 0.06887	2389.8577
BRI	Bari	Pakistan	West Asia	68.86	26.08	25	0.346	0.361	0.086	0.04327	2244.915
BUT	Bugituri	Pakistan	West Asia	68.86	26.08	31	0.355	0.360	0.081	0.01441	2244.915
DDP	Dera Din Panah	Pakistan	West Asia	72.48	30.30	20	0.360	0.367	0.080	0.01994	2389.8577
KAC	Kachan	Pakistan	West Asia	68.86	26.08	19	0.359	0.359	0.116	0.00062	2244.915
KAM	Kamori	Pakistan	West Asia	68.86	26.08	38	0.358	0.352	0.102	− 0.01718	2244.915
LOH	Lohri	Pakistan	West Asia	68.86	26.08	17	0.386	0.371	0.077	− 0.04128	2244.915
PAH	Pahari	Pakistan	West Asia	72.48	30.30	19	0.396	0.386	0.057	− 0.02704	2389.8577
PAT	Pateri	Pakistan	West Asia	68.86	26.08	27	0.367	0.368	0.068	0.00478	2244.915
TAP	Tapri	Pakistan	West Asia	68.36	25.47	22	0.368	0.376	0.067	0.02208	2235.0932
TED	Teddi	Pakistan	West Asia	72.48	30.30	47	0.372	0.372	0.069	0.00155	2389.8577
THA	Thari	Pakistan	West Asia	68.86	26.08	16	0.394	0.390	0.050	− 0.01262	2244.915
NJ	Nanjiang	China	East Asia	82.63	40.70	23	0.390	0.384	0.059	− 0.01778	3167.4685
QG	Qinggeda	China	East Asia	88.23	43.29	24	0.406	0.403	0.038	− 0.00749	3640.5662
AC	Aarbas Cashmere	China	East Asia	108.09	38.79	59	0.382	0.374	0.069	− 0.02258	5351.9616
JN	Jining Grey	China	East Asia	116.27	35.25	39	0.413	0.408	0.049	− 0.01356	6163.9232
LP	Luoping Yellow	China	East Asia	104.45	25.05	24	0.352	0.356	0.116	0.01168	5538.9409
GF	Guangfeng	China	East Asia	118.23	28.32	24	0.341	0.362	0.094	0.06049	6633.596

Distance, distance (in kilometres) between Ganj Dareh and Asian sampling locations calculated with the Vincenty ellipsoid-model method (Ganj Dareh: lon 34.27º, lat 47.47º)

Lon: longitude in degrees; Lat: latitude in degrees; N: sample size of each breed; H_o_: Observed heterozygosity; H_e_: Expected heterozygosity; F_ST_: coefficient of genetic differentiation related to the Iranian Markhoz breed; F_is_: inbreeding coefficient

### Population structure analysis

We assessed population structure using PLINK v. 1.9 [19] to carry out a principal component analysis (PCA) and the R software v.4.1.3. was employed for visualizing the resulting plot. Considering the large number of breeds and samples, the same software was used to plot the centroids of the principal components 1 and 2 for each breed, and such values were used to construct the PCA presented in the main and Additional Figures. Moreover, population structure was investigated with the ADMIXTURE v.1.3.0 package [21] with number of clusters (K) varying from 2 to 15. To assess the quality of the clustering process and thus infer the most likely K-value, we estimated the cross-validation error for each K-value. To visualize the results of the ADMIXTURE analysis, we used the *Pophelper* R package [22].

### Correlating genome-wide diversity with distance to Ganj Dareh

We employed Arlequin v. 3.5.2.2 [23] to calculate observed heterozygosity (H_o_), expected heterozygosity (H_e_), the F_ST_ coefficient of differentiation, and the inbreeding coefficient F_is_. The main reason for calculating both H_o_ and H_e_ is that they provide complementary information: while H_e_ is estimated from allele frequencies, H_o_ is calculated from individual genotypes directly and depends on both the magnitude of genetic diversity in the population and the amount of inbreeding [24]. Moreover, their contrast (F_is_ = 1−$\frac{Ho}{He}$) provides valuable insights about the patterns of variation, with negative and positive values indicating the existence of a deficit (e.g. due to admixture) or an excess (e.g. due to inbreeding) of homozygous genotypes, respectively.

We have chosen Ganj Dareh, in the central Zagros Mountains (Western Iran), as a location representative of the geographic coordinates of the areas of early goat management in the Fertile Crescent, since substantial archaeological and genetic evidence support the practice of goat husbandry in this region at least 10,200 YBP [1, 25]. To calculate geographic distances (in kilometers) from the sampling site of each breed to Ganj Dareh (latitude = 34.27º N and longitude = 47.47º E), we have used the latitude and longitude coordinates provided by Colli et al. [16], Stella et al.[10], and Ouchene-Khelifi et al. [11]. The sampling site lists of the South African, Algerian, Chinese and Iranian populations are available in Chokoe et al. [13], Rahmatalla et al. [12], Berihulay et al. [14], and Nazari-Ghadikolaei et al. [15], respectively, and we have searched for the corresponding coordinates in the open source databases available online (https://www.latlong.net/). For Swiss [17], Irish and British (Old Irish Goat Society, https://oldirishgoat.ie) breeds, we used centroids of country geographic coordinates to calculate distances to Ganj Dareh since the coordinates of sampling sites were not available. Geographical distances were obtained with the *geosphere* package [26] of the R software v.4.1.3. using the “*distVincentyEllipsoid*” method which considers the earth as an ellipsoid flattened at the poles, thus providing a very accurate calculation of distances [27]. We estimated pairwise F_ST_ coefficients between the Iranian Markhoz breed, which is raised in an area (Latitude = 35.32º N and Longitude = 46.98º E) close to Ganj Dareh, against all population from Europe, Africa, and Asia. Pearson correlation coefficients (*r*) were computed to assess if there is a linear relationship between H_o_, H_e_ and F_ST_ estimates and geographical distances between breed sampling sites and Ganj Dareh by using the *stats* package included in the R software v.4.1.3 [28]. Linear regressions were plotted with the *ggplot2* package of R software v.4.1.3. For Europe and Africa, we did two separate analyses including or excluding insular populations. The reason for not including insular populations is that they usually have reduced levels of diversity due to geographic isolation rather than to ancient post-domestication events [29]. In the case of African populations, we excluded from our analysis goats from the Boer, Savanna, and Kalahari Red breeds because there is evidence that their ancestry has an Asian component, so they are not fully representative of South African indigenous local goats [16, 30].

In addition, H_o_, H_e_ and F_is_ values computed for each population were used to construct interpolation maps drawn using the inverse distance weighted (IDW) option implemented in the GIS software ArcGIS v. 3.2.0 (https://www.arcgis.com/index.html ESRI, Redlands, CA, United States). This deterministic method of multivariate interpolation considers a set of scattered points with known values for a variable and calculates the values of the variable for points with missing values by taking into account the weighted average of the values available at the known points. The measured values closest to the location to be predicted have more influence on the predicted value than those farther away. The sampling area of each population was used as geographic coordinates and interpolation surfaces were divided into ten equal classes. Moreover, to evaluate whether inbreeding could affect our inferences about the potential existence of gradients of diversity, we have retrieved all F_ROH_ values from goat breeds reported by Bertolini et al. [31] in the framework of the AdaptMap project (as long as their sample sizes were above 15 individuals). Then, we have calculated Pearson correlations between such coefficients and distance to Ganj Dareh.

## Results and discussion

### Population structure and global diversity analysis

**Fig. 1:**
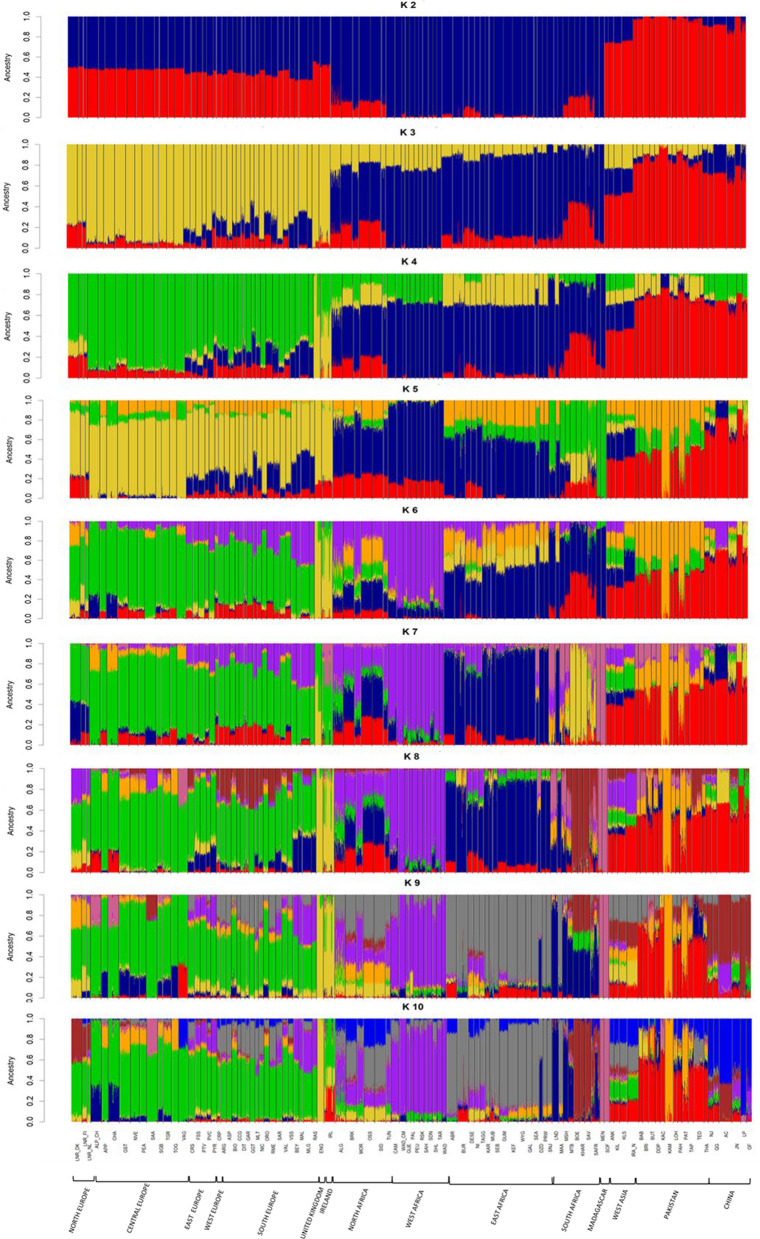

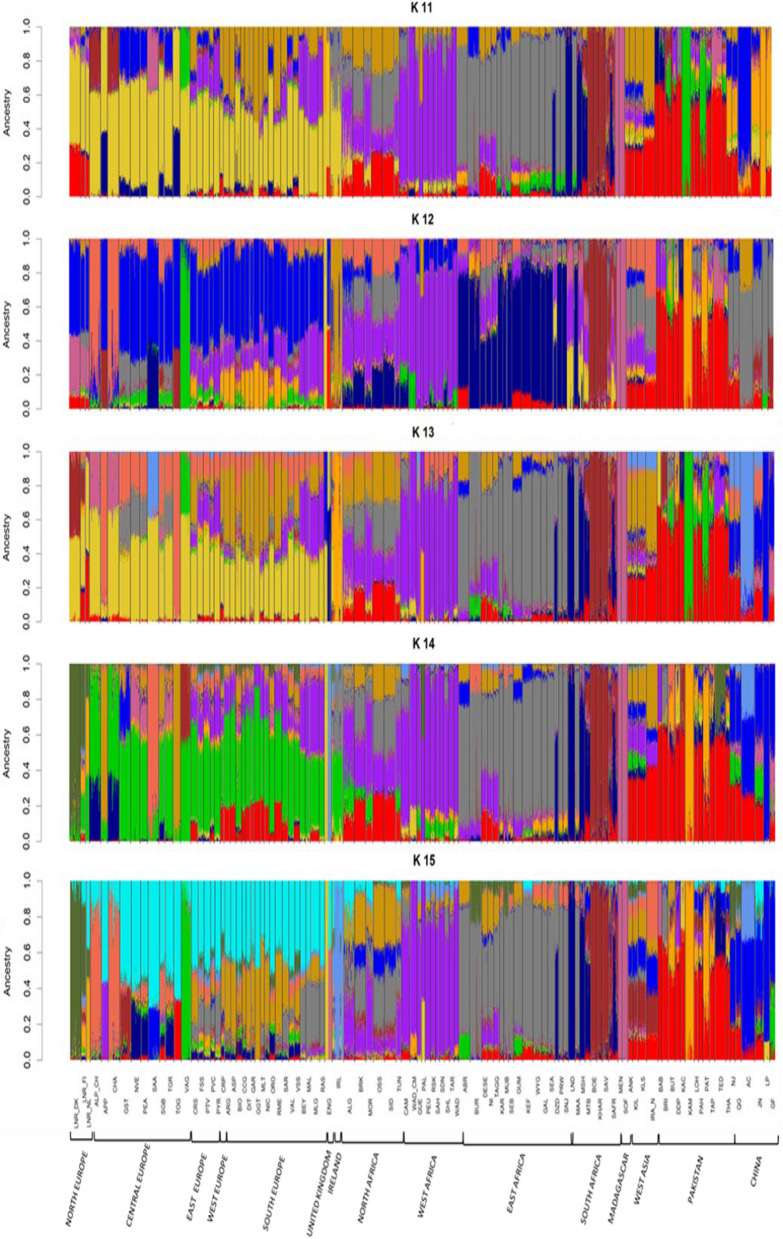
ADMIXTURE analysis of African, European Asian goat breeds included in our study. Each bar represents the percentages of global ancestries from one or more of K = 2–15 genetically distinct sources for each individual. Continental subregions in Africa include the following countries: (1) Northern Africa: Morocco, Algeria, Tunisia, and Egypt, (2) Western Africa: Burkina Faso, Mali, Nigeria, Cameroon, and Canary Islands, (3) Eastern Africa: Sudan, Ethiopia, Uganda, Burundi, Kenya, Tanzania, and Malawi, (4) Southern Africa: Mozambique, Zimbabwe, and South Africa, and (5) Madagascar. Continental subregions in Europe include the following countries: (1) Northern Europe: Denmark, Finland, and The Netherlands, (2) Central Europe: Switzerland, (3) Western Europe: France, (4) Eastern Europe: Romania, (5) Southern Europe: Italy and Spain, and (6) United Kingdom and Ireland. Continental subregions in Asia include the following countries: (1) West Asia: Iran and Turkey, (2) Pakistan and (3) China. African, European and Asian breeds and subregions names are reported

**Fig. 2:**
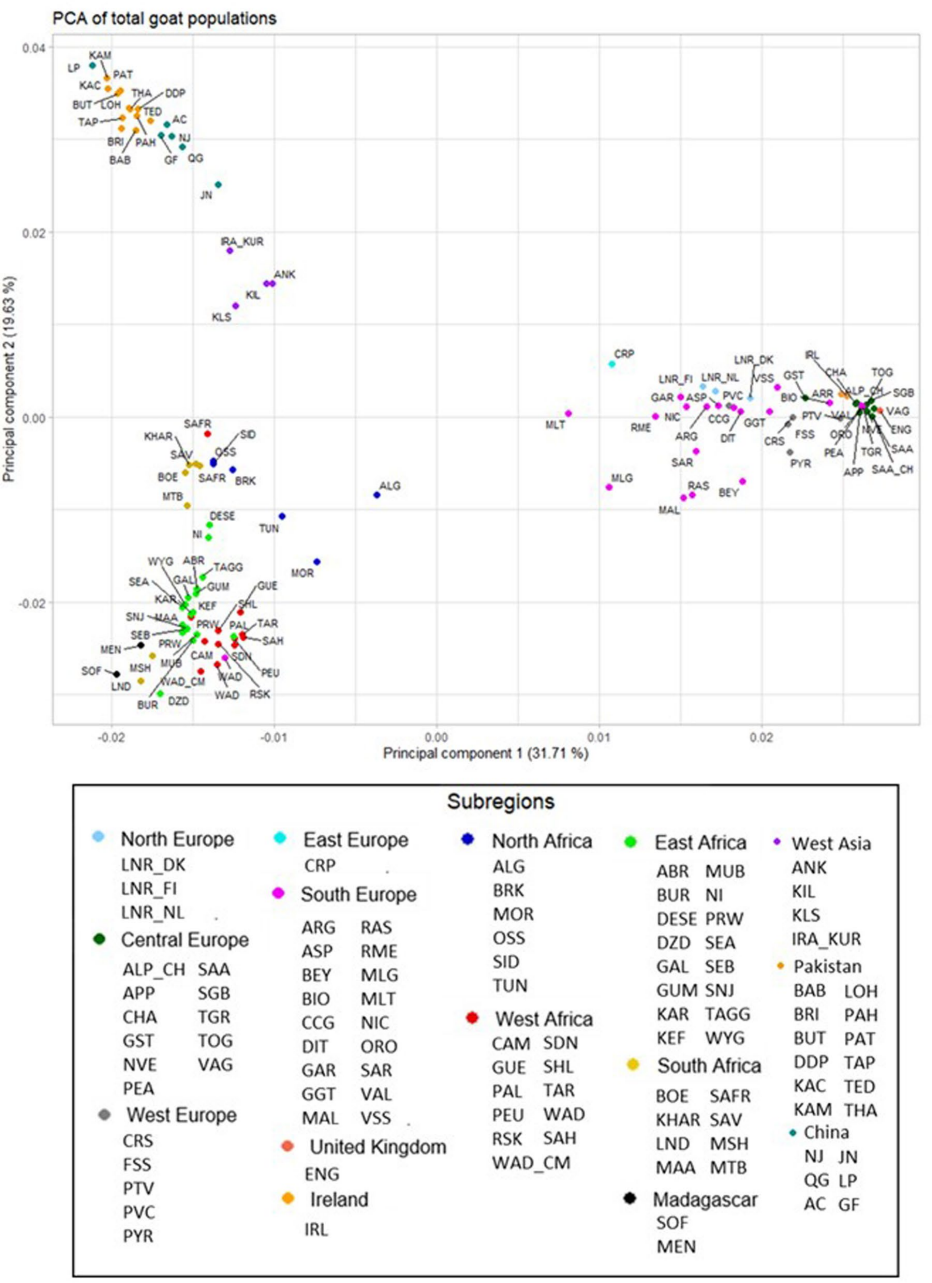
Principal Component Analysis plot of European, African and Asian breeds. Principal components 1 and 2 and percentages of variance explained by them. The figure shows the centroids of principal components 1 and 2 for each breed. Samples are coloured according to their continental subregion of sampling and represented by breed acronyms. The list of complete breed names can be found in Tables 1, 2 and 3

We analyzed the population structure of the European, African and Asian goats by using ADMIXTURE (Fig. 1) and PCA (Fig. 2) tools. Regarding European breeds, we observed a partial regional differentiation, except for those from Northern Europe (Denmark, The Netherlands and Finland), Great Britain and Ireland. Strong differences in autosomal SNP as well as chromosome Y haplotype frequencies have been observed when comparing Northern and Southern European goats [16, 30] and we have detected the same trend in the PCA shown in Additional file 1 Figure S2, with the 50º latitude dividing Northern and Southern European goats. This pattern can be explained partially by the post domestication dispersal of goats across Europe through two main corridors: the Mediterranean route, which involved the maritime transportation of livestock along the Mediterranean basin until reaching the Iberian Peninsula 7300–7700 YBP, and the Danubian route, which traversed the European mainland and reached Scandinavia and the British Isles 4000 YBP [3].

For African goats, we have observed five main clusters representing populations from South, West, North and East Africa plus a fifth Malagasy group (see Additional file 1: Figure S3), which was supported by the ADMIXTURE analysis (Fig. 1) and agrees with previous findings [16]. Geographic (e.g. Sahara and Kalahari deserts) and biological (e.g. Tsetse fly belt) barriers may have contributed substantially to the genetic differentiation of goat populations from West, East, North and South Africa. In the case of Malagasy goats, their genetic differentiation from continental populations is probably explained by their insular origin and the likely occurrence of a strong founder effect [29]. Finally, Palmera goats cluster with the West African breeds because they were transported to the Canary Islands by settlers of Amazigh origin 2000–2500 YBP [32].

In the case of Asian goats (see Additional file 1: Figure S4), we can observe three main clusters represented by goats from West Asia/Near East (Turkey and Iran), South Asia (Pakistan) and East Asia (China). The early diffusion of goat pastoralism in Asia has not been characterized in depth yet, but Pereira and Amorim [33] have proposed two main corridors of dispersal, i.e. (1) the central Asian steppes, traversing Afghanistan and reaching Mongolia and northern China, and (2) through the Indus Valley spreading into the Indian subcontinent and, subsequently, to Southeast Asia. Interestingly, the analysis of archaeological remains at the Djeitun site in Southern Turkmenistan dated to ca. 8500 YBP provided evidence about the important role of ovicaprids as a source of animal protein [34]. Besides, more recently, zooarchaeological and collagen peptide mass fingerprinting demonstrated the ancient husbandry of sheep and goats at the Obishir V site in Southern Kyrgyzstan 8000 YBP [34]. Moreover, evidence dating back to 4912–4761 YBP has been acquired, indicating the consumption of milk from sheep and other unidentified ruminants among Afanasievo groups in the Altai mountains [35]. These mountains serve as a natural boundary, separating the lowlands of Kazakhstan and Western Siberia from Mongolia. The entry of goats in China might have taken place through the Hexi Corridor (Gansu-Qinghai region, 5600–5000 YBP), and/or by crossing the Eurasian steppes and the Mongolian Plateau (∼5500–4500 cal YBP) [36]. This complex process of pastoralism diffusion in Asia, which is still quite unknown, might have led to the establishment of highly differentiated goat gene pools in the three regions (West, South and East Asia) under study, as shown in Additional file 1: Figure S4.

The global average values of H_o_ and H_e_ for European (H_o_ = 0.394, H_e_ = 0.393), African (H_o_ = 0.391, H_e_ = 0.393) and Asian (H_o_ = 0.381, H_e_ = 0.381) populations were quite similar and, in general, high. The average F_is_ coefficients, that indicate the departure of H_o_ from H_e_, were − 0.0025, 0.0033 and − 0.0012 for European, African and Asian populations, respectively. With regard to F_ROH_ values (see Additional file 2: Table S1), a low average coefficient (F_ROH_ = 0.08) was estimated for European breeds, with the highest value for the northern European Landrace breed (F_ROH_ = 0.16). Similarly, for the African breeds, a low average F_ROH_ value (F_ROH_ = 0.08) was estimated, with the highest coefficients for populations from Madagascar (Sofia: F_ROH_ = 0.35; Menabe: F_ROH_ = 0.32) and the Palmera breed of the Canary Islands (F_ROH_ = 0.23). In contrast, a moderate average F_ROH_ value (F_ROH_ = 0.13) was found for Asian goats, with F_ROH_ values of 0.25 for the Kachan and Kamori breeds from Pakistan.

### Diversity of European goat populations is not correlated with distance to Ganj Dareh

**Fig. 3:**
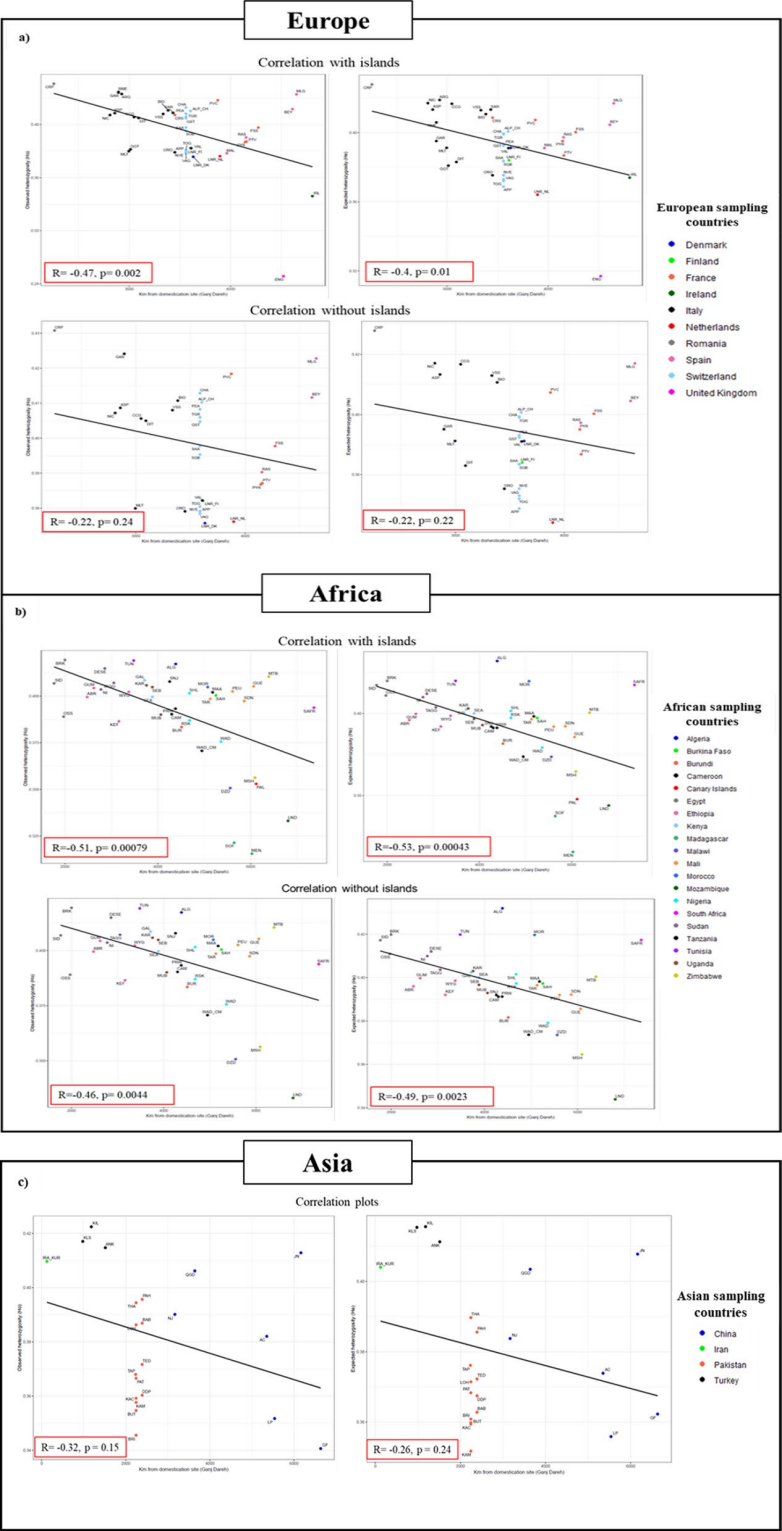
Graphs depicting the relationships between observed and expected heterozygosities of European, African and Asian goat populations and distance between their sampling locations and Ganj Dareh. Graphs depicting the relationships (expressed as Pearson correlations and their *P*-values) between observed heterozygosity and expected heterozygosity and distance from Ganj Dareh (early Neolithic settlement in the Zagros Mountains representative of the geographic coordinates of the areas of early goat management in the Fertile Crescent) to sampling locations of **a** European breeds, including and not including insular populations, **b** African breeds, including and not including insular populations, **c** Asian populations. In all plots, country of origin is indicated with specific colours. Breed acronyms are listed in Tables 1, 2 and 3

We investigated whether H_o_ and H_e_ values of African, European, and Asian populations show significant correlations (r) with distance from their sampling location to Ganj Dareh. When analyzing goat populations from Europe (Fig. 3a), we obtained negative and significant correlations (H_o:_ r =  − 0.47, *P* = 0.002, Fig. 3a; He: r = − 0.40, *P* = 0.01, Fig. 3a) for both heterozygosity values. However, these two correlations became non-significant (H_o_: *r* = − 0.22, *P* = 0.24, Fig. 3a; H_e_: *r* = − 0.22, *P* = 0.22, Fig. 3a) when British and Irish populations were removed from the European data set. Indeed, the majority of European breeds displayed moderate to high heterozygosity values (Fig. 3a), with the exception of the populations from United Kingdom (H_o_ = 0.29; H_e_ = 0.32) and Ireland (H_o_ = 0.35; H_e_ = 0.37). Even the Spanish Bermeya and Malagueña breeds, which are located very far apart from Ganj Dareh, displayed high heterozygosities (H_o_ = 0.41; H_e_ = 0.40 in Bermeya and H_o_ = 0.42; H_e_ = 0.42 in Malagueña). On the other hand, correlations between F_ST_ values and distance to Ganj Dareh were positive and significant when insular populations were included in the analysis (*r* = 0.37, *P* = 0.02), but became non-significant (*r* = 0.28, *P* = 0.12) when such populations were removed from the analysis (see Additional file 1: Figure S5a). Moreover, correlations between F_ROH_ values and distance to Ganj Dareh with (*r* = 0.13, *P*-value = 0.52) or without (*r* = 0.06, *P*-value = 0.79) islands were non-significant (see Additional file 1: Figure S6a), and the interpolation map (see Additional file 1: Figure S7) and list (see Table 1) of F_is_ values evidenced that they are, in general, weak and negative.

Such results do not fully match those of Cañón et al. [7], who described a decrease in caprine genetic diversity from the south-east to the north-west of Europe. This could be due to the limited number of microsatellite markers used by Cañón et al. [7], but also to the fact that Cañón et al. [7] had a much broader collection of Eastern European goat breeds than us. The significant gradient that we observe when British and Irish populations are included in the analysis might be due to their strong demographic recession [37], which is reflected by their high levels of homozygosity [29]. However, we cannot rule out the possibility that the low diversity of British and Irish cattle is partly explained by one or more founder effects associated with the arrival of livestock to the United Kingdom and Ireland 5800–6000 YBP, as suggested for British cattle [38].

The lack of a significant gradient of diversity in European goat breeds could be due to post-domestication migratory movements associated with trading and herding. Throughout the millennia, the Mediterranean Sea has facilitated the exchange of goods and livestock via a dense network of commercial maritime routes connecting distant port cities within and outside Europe. Indeed, Cardoso et al. [29] reported that goats from Mediterranean islands have lower levels of homozygosity than those from remote islands as Iceland, La Palma or Madagascar. In addition, the Great European Plain, which is one of the largest continuous expanses of plain on the Earth's surface, may have facilitated the exchange of goats and other livestock amongst distant locations within Europe. This interpretation is supported by the mostly negative F_is_ values shown in the corresponding interpolation map (see Additional file 1 Figure S7), which are compatible with a slight excess of heterozygosity. Even more, in recent times the widespread use of improved breeds (e.g., Saanen, Toggenburg and Alpine), and artificial insemination might also have contributed to increasing gene flow between distant European populations. Besides, there is evidence that these highly productive cosmopolitan breeds have introgressed many local breeds in Europe [30].

### Detection of a significant gradient of diversity associated to distance to Ganj Dareh in African goats

In contrast with European goats, significant negative correlations between the diversity of African caprine populations and distances to Ganj Dareh have been observed in the data sets with (Madagascar and La Palma) and without islands (Fig. 3b). Indeed, we obtained correlation coefficients of − 0.46 (H_o_, *P* = 0.0044) and − 0.49 (H_e_, *P* = 0.0023) in the data set with no islands (Fig. 3b**)** and correlation coefficients of − 0.51 (H_o_, *P* = 0.00079) and − 0.53 (H_e_, *P* = 0.00043) in the data set with islands (Fig. 3b). Consistently, the magnitude of F_ST_ coefficients was highly correlated with distance from the African sampling sites to Ganj Dareh for both data sets with (*r* = 0.57, *P* = 0.00011) and without (*r* = 0.62, *P* = 0.000045) islands (see Additional file 1: Figure S5b). The correlation between F_ROH_ and distance to Ganj Dareh was not significant (*r* = 0.23, *P* = 0.21) when insular populations were excluded from the analysis, while it became significant (r = 0.38, *P* = 0.025) when Malagasy goats were taken into consideration (see Additional file 1: Figure S6b). This result could be anticipated because Malagasy goats have high F_ROH_ coefficients, probably because of the occurrence of a strong founder effect [29]. Based on these results and the interpolation map (see Additional file 1: Figure S7) and list (See Table 2) displaying F_is_ values, which are mostly close to zero and negative (except in North Africa), we conclude that the decrease of diversity associated to distance to Ganj Dareh observed in African breeds is not caused by a parallel augment of inbreeding.

We have observed that the Egyptian, Algerian, and Sudanese populations, which are closest to the Fertile Crescent, show the highest heterozygosity values (see Table 2). When proceeding southwards and particularly south-eastwards, diversity decreases, as evidenced in goat breeds from Mozambique (H_o_ = 0.33; H_e_ = 0.34) and Malawi (H_o_ = 0.35; H_e_ = 0.37), and particularly in the island of Madagascar (H_o_ = 0.31; H_e_ = 0.33). With regard to indigenous South African breeds, their diversity is high (H_o_ = 0.39; H_e_ = 0.42), probably because many of these breeds have been introgressed by Boer goats. The Boer breed has a mixed Asian and African ancestry [30], and there is evidence that Anglo-Nubian bucks contributed to its foundation [4].

The dispersal of livestock by land is expected to take place through a series of founder effects, thus generating gradients of decreasing diversity and increasing genetic differentiation as the ones observed in our work. In contrast, when domestic animals are transported by sea it is more likely to observe a leap-frog pattern of diffusion that does not necessarily result in genetic clines of differentiation or diversity. In consequence, the detection of a gradient of diversity (H_o_ and H_e_) and genetic differentiation (F_ST_) associated with distance to Ganj Dareh in African goats is consistent with an overland rather than maritime post-domestication dispersal of goats throughout the African continent, with the only exception of the North African shoreline where maritime diffusion throughout the Mediterranean Sea was important [39] as attested by remains of impressed pottery, crop plants and sheep, goats, and cattle remains found in archaeological sites in Lybia, Algeria and Morocco [40]. The predominant overland spread of domesticates in Africa (when compared to Europe) might be explained by the fact that the surfaces of Europe and Africa are about 10 million km^2^ and 30 million km^2^, respectively, while their coastal lines are 30,000 km (Africa) and 143,000 km (Europe) long [41, 42]. Besides, in Africa there is a relative scarcity of natural harbors and long navigable river systems, the latter due to the ruggedness of the terrain, with rapids and waterfalls as well as shallow river points, strong seasonal fluctuations in water flow, siltation, and sedimentation in lower reaches [41]. This means that the inner parts of the African continent are less easily accessible by navigation than European inland, making transportation of livestock difficult.

The early entry of goats in Africa probably took place in North Africa through the Sinai Peninsula as well as through the Mediterranean Sea [6], coinciding with the opening of a grassland niche in the Sahara that was gradually occupied by pastoral communities [6]. The increasing aridity of the Sahara around 4500 YBP and the consequent southward retreat of the Tsetse fly belt favored the migration of herders towards the Sahel. However, the entry of livestock into West and East Africa took place not before than 3500 YBP or even later [40], possibly because of a lack of immunity to endemic diseases. Goat and sheep remains dating back to 2400 YBP and 2100 YBP have been found at the sites of Salumano (Zambia) and Bamba (Zimbabwe), proving that the arrival of small ruminants to Southern Africa is quite recent [6]. This might have involved migrations through and along the coastal areas of the Congo Basin or facilitated by the opening of Tsetse corridors along the highland of the Rift Valley [40, 43]

**Fig. 4:**
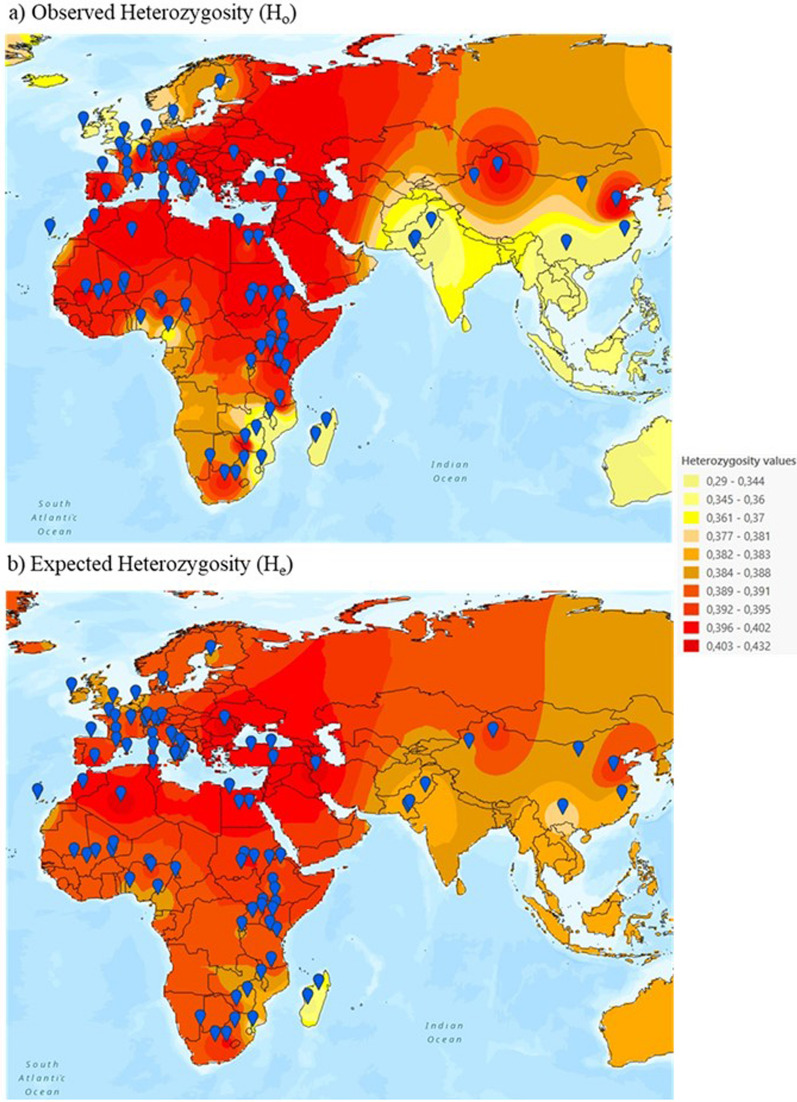
Interpolation maps showing the geographic distribution of observed and expected heterozygosities in African, European and Asian breeds. Interpolation maps showing the distribution of genetic diversity in African, European and Asian breeds. **a** Observed heterozygosity, H_o_. **b** Expected heterozygosity, H_e_. Blue points represent sampling localities in **a** and **b**, respectively. In Europe, a reduction of diversity is evident in goats from the United Kingdom and Ireland, while in Africa low diversity coincides with the Tsetse fly belt (a geographic area comprised between latitudes 14° N and 20° S) and Madagascar. In Asia, low variation is detected in Pakistan and Southern China

As shown in Fig. 4, goats from Central and East Africa are less diverse than their Northern counterparts, possibly because the Sahara Desert, which covers 9.1 million km^2^, constitutes a formidable geographical barrier to the southwards spread of pastoral communities and their livestock [44]. Moreover, Central Africa overlaps with the Tsetse fly belt, which covers a geographic area of 10 million km^2^, between latitudes 14° N and 20° S, representing about one third of the African continent. Trypanosomiasis is a protozoan disease which causes anemia, fever, and weight loss and sometimes can be fatal, representing a heavy economic burden to African countries in which this infection is endemic [45]. Susceptibility to this parasite may have limited the diffusion and exchange of caprine stocks in Tsetse fly infested areas. Interestingly, Traorè and coworkers showed that the presence of the Tsetse fly influences the genetic variability of goats from Burkina-Faso, and they demonstrated that trypanosomiasis might have acted as a landscape boundary both for the spread of trypanosensitive goats and for strong selection pressure on trypanotolerant goats in infested areas [46].

We have detected a high variability of several South African indigenous breeds even though this region remained considerably isolated from Asia and Europe [47]. We excluded from the gradient analysis South African commercial goats (Boer, Kalahari Red and Savanna) because it is well known that Boer goats have a mixed African and Asian ancestry [16, 30], and that Kalahari and Savanna goats have a strong Boer component. We kept in our analysis indigenous communal populations sampled in the main goat-producing provinces of South Africa (Limpopo, Freestate, Gauteng, Northwest), which happened to have high levels of heterozygosity. This could be due to the fact that these South African populations have been also introgressed to some extent by Boer goats as well as by goats of European origin. Indeed, the establishment, in South Africa, of British and Dutch farmers, during the seventeenth-nineteenth centuries, promoted the development or importation of highly productive breeds to improve the local stocks [4].

### Absence of a gradient of caprine diversity in Asia

In the case of the Asian goat breed data set (which does not include insular breeds), we obtained correlation coefficients of − 0.32 (H_o_, *P* = 0.15; Fig. 3c) and − 0.26 (H_e_, *P* = 0.24, Fig. 3c) when contrasting heterozygosity values against distance to Ganj Dareh, while the correlation between such distance and F_ST_ values (r = 0.24, *P* = 0.30) was also non-significant (see Additional file 1: Figure S5c). Moreover, when we investigated the correlation between F_ROH_ and distance to Ganj Dareh (see Additional file 1: Figure S6c), we obtained a significant and positive value (r = 0.60, *P* = 0.02). This latter analysis only encompassed AdaptMap populations from Turkey and Pakistan, so the number of observations is relatively limited. However, the inspection of Additional file 1: Figure S6c makes evident that goat breeds from Pakistan display a range of F_ROH_ values considerably broader than those observed in European or African continental populations. The interpolation map (see Additional file 1: Figure S7) and list (Table 3) showing F_is_ values also evidenced that in Asian goats such coefficients are slightly positive, a potential indication about the existence of inbreeding. Kumar et al. [48] examined the diversity of seven indigenous Pakistani goat populations and found that five of them (Bari, Black Tapri, Bugitoori, Kamori and Pateri) displayed F_ROH_ values close to or above 0.10, with the Bugitoori breed being particularly inbred (F_ROH_ = 0.34). Information about the history and demography of the Pakistani breeds investigated in our study is very scarce, so it is difficult to disentangle why several of them have such high inbreeding coefficients. One potential reason would be the occurrence of series of floods (about 1 million of domestic animals were killed in 2022 floods), prolonged and extreme periods of drought, and severe heat waves which have caused significant losses of livestock resources in several places in Pakistan, including Punjab which is the most important agricultural area of the country [49]. We hypothesize that such abrupt demographic reductions might have led to increases in inbreeding levels of goat populations from the affected areas, although we cannot rule out other alternate explanations.

## Conclusions

A genetic cline associated with distance to Ganj Dareh has been observed in African goats but not in their European and Asian counterparts. Regarding Asian goats, we have just sampled goat breeds from four countries, so it is difficult to anticipate whether a more extensive sampling could lead to the detection of such genetic cline. In the case of African goats, the existence of a gradient of diversity could be explained, at least in part, by a predominantly overland post-domestication dispersal of goats in Africa due to the paucity of natural harbors and navigable rivers in this continent. In contrast, Europe has a long coastline, a feature that might have favored the maritime diffusion of the Neolithic package. Besides, about two thirds of the African continent are occupied by two formidable geographic (Sahara Desert) and biological (Tsetse fly belt) barriers that restrict the long-distance transportation of livestock, while most of Europe is covered by an uninterrupted plain that goes from the Pyrenees to the Ural Mountains. In this context, it is reasonable to assume that the migratory movements of goats (and other livestock), since domestication to present, were more intense, sustained, and recurrent in Europe than in Africa, a circumstance that might have enhanced the erasure of any genetic signature left by the initial spread of domesticates. The combination of these and other factors might explain why a post-domestication gradient of diversity is still detectable in African goats but not in their European counterparts.

### Supplementary Information

Additional file 1: Figure S1. Geographic distribution of (a) European, (b) African and (c) Asian goat breeds. It shows the geographic locations and acronyms corresponding to all goat populations under study, which have been coloured according to their country of origin. Figure S2. Principal Component Analysis (PCA) plot of the first two components for 38 European goat breeds. We reported the centroids of principal components 1 and 2 for each breed under study. The percentages of variation explained by the two main components of the PCA are shown in brackets. Individuals are coloured according to their subregion of sampling. Breed acronyms are as follows: ALP_CH= Alpine, APP=Appenzell, ARG= Argentata, ASP= Aspromontana, BEY= Bermeya, BIO= Bionda dell’Adamello, CCG= Ciociara Grigia, CHA= Swiss Chamois, CRP= Carpathian goat, CRS= Corse, MAL= Mallorquina, MLG= Malagueña, MLT=Maltese, NIC= Nicastrese, NVE= Nera Verzasca, ORO= Orobica, PEA= Peacock, PTV= Poitevine, PVC= Provençale, PYR= Pyrenean, DIT= Di Teramo, ENG= Old English Goat, FSS= Fosses, GAR= Garganica, GGT= Girgentana, GST= Grisons striped, IRL= Old Irish Goat, LNR_DK= Landrace Goat (Denmark), LNR_FI= Landrace Goat (Finland), LNR_NL= Landrace Goat (Netherlands), RAS= Blanca de Rasquera, RME= Rossa Mediterranea, SAA= Saanen, SAR= Sarda, SGB= Booted goat, TGR= Tessin grey goat, TOG= Toggenburg, VAG= Valais, VAL= Valdostana,VSS= Valpassiria. Figure S3. Principal Component Analysis (PCA) plot of the first two components for 43 African goat breeds. We reported the centroids of principal components 1 and 2 for each breed under study. The percentages of variation explained by the two main components of the PCA are shown in brackets. Individuals are coloured according to their subregions of sampling. Breed acronyms are as follows: ABR= Abergelle, ALG= Arabia,Makatia, and M'Zabite,Kabyle, BOE=Boer, BRK=Barki, BUR= Burundi goat, CAM= Cameroon Goat, DESE=Desert, DZD= Dedza, MAA= Maasai, MEN= Malagasy goat (Menabe), MOR= Barcha,Draa,Ghazalia, Moroccan goats, Noire de l'Atlas,Nord, MSH= Mashona, MTB= Matebele, MUB= Mubende, NI= Nilotic, OSS= Oasis, SDN= Soudanaise, SEA= Small East African, SEB= Sebei, SHL= Sahel, SID= Saidi, SNJ=Sonjo, SOF= Malagasy goat (Sofia), TAGG= Taggar, GAL= Galla, GUE= Guera, GUM= Gumez, KAR= Karamonja, KEF= Keffa, KHAR= Kalahari Red, LND= Landin, PAL= Palmera, PEU= Peulh, PRW= Pare White, RSK= Red Sokoto, SAFR= South African Local breeds (from Limpopo, Freestate,Gauteng,Nortwest), SAH= Sahel, SAV= Savanna. TAR= Targui, TUN= Tunisian, WAD_CM= West African Dwarf (Cameroon), WAD= West African Dwarf (Nigeria), WYG= Woyito Guji. Figure S4. Principal Component Analysis (PCA) plot of the first two components for 22 Asian goat breeds. We reported the centroids of principal components 1 and 2 for each breed under study. The percentages of variation explained by the two main components of the PCA are shown in brackets. Individuals are coloured according to their subregions of sampling. Breed acronyms are as follows: ANK= Ankara (Turkey), KIL= Kol (Turkey), KLS= Kilis (Turkey), IRA_KUR= Markhoz (Iran), BAB= Barbari (Pakistan), BRI= Bari (Pakistan), BUT= Bugituri (Pakistan), DDP= Dera Din Panah (Pakistan), KAC= Kachan (Pakistan), KAM= Kamori (Pakistan), LOH= Lohri (Pakistan), PAH= Pahari (Pakistan), PAT= Pateri (Pakistan), TAP= Tapri (Pakistan), TED= Teddi (Pakistan), THA= Thari (Pakistan), NJ= Nanjiang (China), QG= Qinggeda (China), AC= Aarbas Cashmere (China), JN= Jining Grey (China), LP= Luoping Yellow (China), GF= Guangfeng (China). Figure S5. Correlation graph between the distance (km) from Ganj Dareh to sampling locations of European, African and Asian goat breeds in relation to their F_ST_ values with regard to the Iranian Markhoz population. We report plots representing Pearson correlations (with their *P*-values) between F_ST_ values (differentiation between all populations and the Iranian Markhoz population) and distance between Ganj Dareh and sampling location of (a) European breeds (not including and including insular breeds); (b) African populations (not including and including insular populations); and (c) Asian populations. Breed acronyms can be found in Tables 1, 2 and 3, and the country of origin of goats is indicated with coloured points. Figure S6. Correlation plot depicting the relationship between the distance (measured in kilometers) from Ganj Dareh and the sampling locations of European, African, and Asian goat breeds, with regard to their F_ROH_ values. We report graphs representing Pearson correlations (with their *P*-values) between F_ROH_ coefficients and distance from Ganj Dareh to sampling locations of (a) European breeds (not including and including insular breeds); (b) African populations (not including and including insular populations); and (c) Asian populations. Breed acronyms can be found in Tables 1, 2 and 3, and the country and subregions of origin of goats is indicated with coloured points. Figure S7. Interpolation map of F_is_ values measured in European, African and Asian goat populations. Interpolation map showing the distribution of inbreeding coefficient F_is_ in African, European and Asian breeds. Red points represent sampling localities. In Europe we mostly observe negative and low F_is_ values, as well as in Africa, except for the North where positive values are noticeable (presence of an intense blue colour). In Asia, especially in the south we also have positive values, a potential signature of inbreeding.

Additional file 2: Table S1. List of European, African and Asian goat breeds of the Adaptmap data set and their heterozygosity (H_o_ and H_e_) and F_ROH_ values. F_ROH_ values were calculated by Bertolini et al. (2018) for the set of AdaptMap goat populations. Breed code including breed name, subregions of provenance, observed heterozygosity (H_O_), expected heterozygosity (H_e_) values, average fraction of the genome that contains ROH (F_ROH_) and distance (km) from Ganj Dareh.

## Data Availability

All relevant data are included in the manuscript and in the additional files. Genotype data sets can be accessed at: https://bridgeurl.com/goat-genotype-data-petretto-et-al-2024
