# Supplementary material for: Investigating the footprint of post-domestication dispersal on the diversity of modern European, African and Asian goats
Source: Genet Sel Evol. 2024 Jul 27;56:55. doi: 10.1186/s12711-024-00923-5 (PMC11282621; doi:10.1186/s12711-024-00923-5)
Supplement: Supplementary file 1 — Additional file 1: Figure S1. Geographic distribution of (a) European, (b) African and (c) Asian goat breeds. It shows the geographic locations and acronyms corresponding to all goat populations under study, which have been coloured according to their country of origin. Figure S2. Principal Component Analysis (PCA) plot of the first two components for 38 European goat breeds. We reported the centroids of principal components 1 and 2 for each breed under study. The percentages of variation explained by the two main components of the PCA are shown in brackets. Individuals are coloured according to their subregion of sampling. Breed acronyms are as follows: ALP_CH= Alpine, APP=Appenzell, ARG= Argentata, ASP= Aspromontana, BEY= Bermeya, BIO= Bionda dell’Adamello, CCG= Ciociara Grigia, CHA= Swiss Chamois, CRP= Carpathian goat, CRS= Corse, MAL= Mallorquina, MLG= Malagueña, MLT=Maltese, NIC= Nicastrese, NVE= Nera Verzasca, ORO= Orobica, PEA= Peacock, PTV= Poitevine, PVC= Provençale, PYR= Pyrenean, DIT= Di Teramo, ENG= Old English Goat, FSS= Fosses, GAR= Garganica, GGT= Girgentana, GST= Grisons striped, IRL= Old Irish Goat, LNR_DK= Landrace Goat (Denmark), LNR_FI= Landrace Goat (Finland), LNR_NL= Landrace Goat (Netherlands), RAS= Blanca de Rasquera, RME= Rossa Mediterranea, SAA= Saanen, SAR= Sarda, SGB= Booted goat, TGR= Tessin grey goat, TOG= Toggenburg, VAG= Valais, VAL= Valdostana,VSS= Valpassiria. Figure S3. Principal Component Analysis (PCA) plot of the first two components for 43 African goat breeds. We reported the centroids of principal components 1 and 2 for each breed under study. The percentages of variation explained by the two main components of the PCA are shown in brackets. Individuals are coloured according to their subregions of sampling. Breed acronyms are as follows: ABR= Abergelle, ALG= Arabia,Makatia, and M'Zabite,Kabyle, BOE=Boer, BRK=Barki, BUR= Burundi goat, CAM= Cameroon Goat, DESE=Desert, DZD= Dedza, MAA= Maasai, MEN= Malagasy goat (Menabe), MOR= B [file 12711_2024_923_MOESM1_ESM.pptx]

## Slide 1
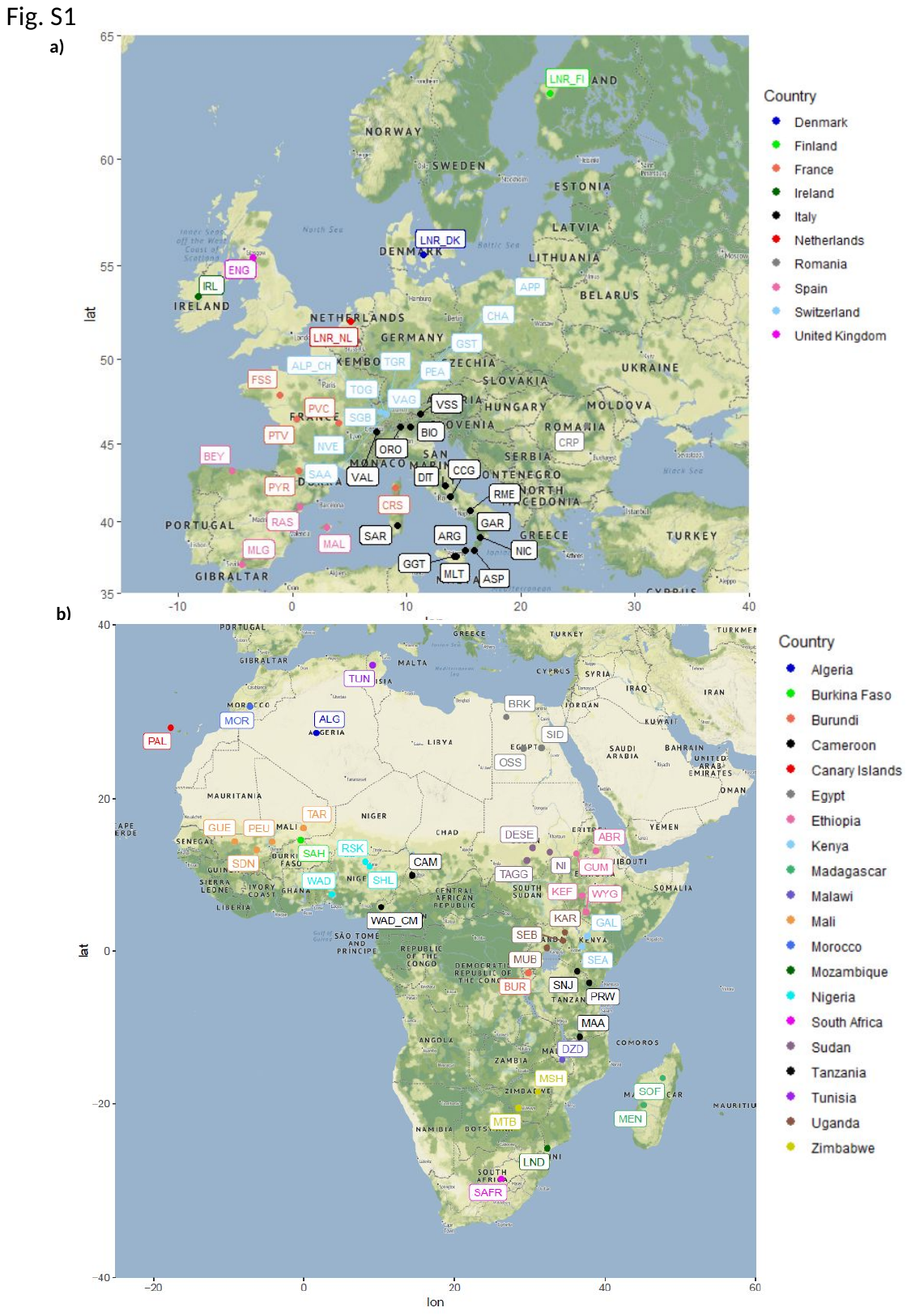

Fig. S1
a)
b)

## Slide 2
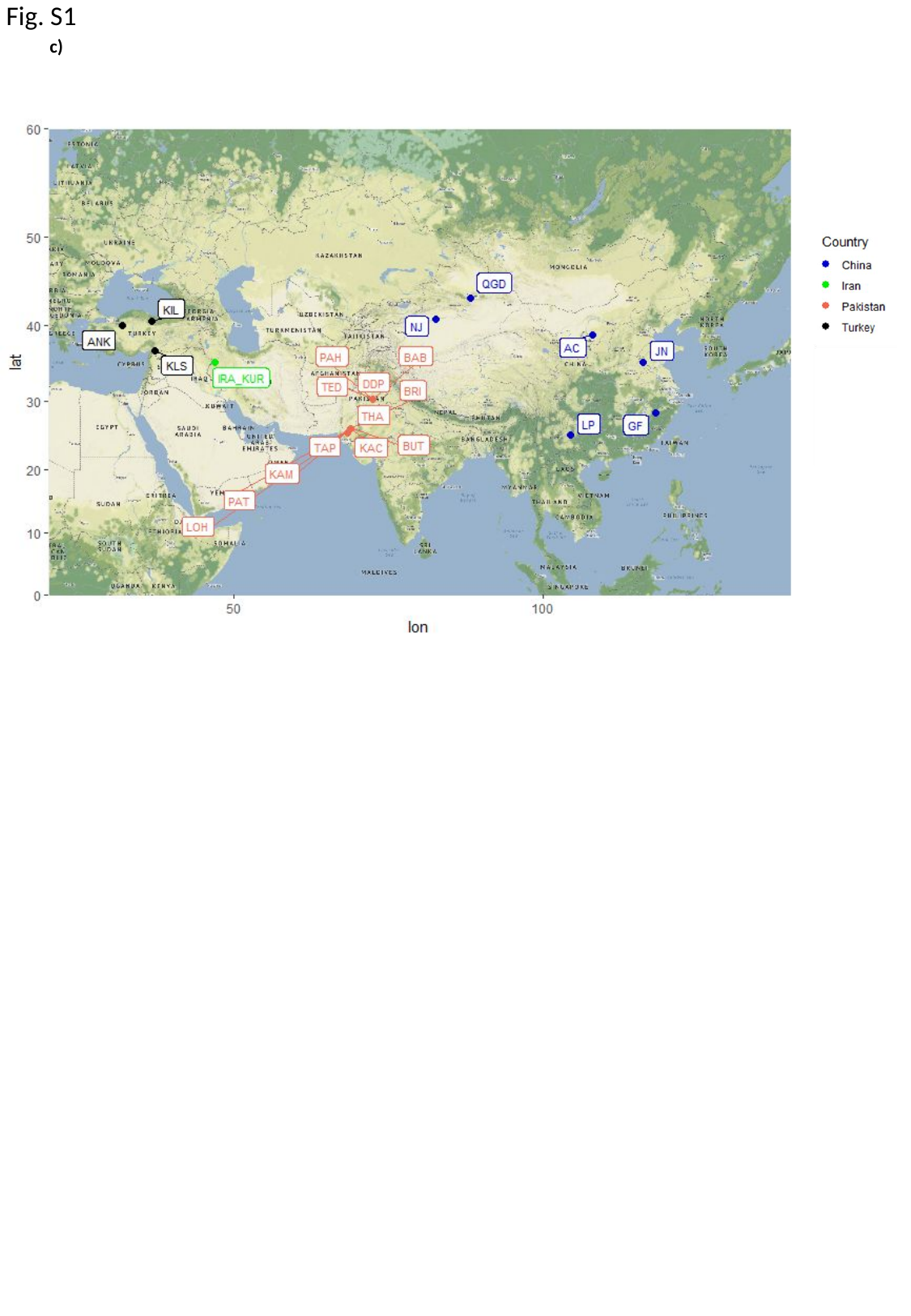

Fig. S1
c)

## Slide 3
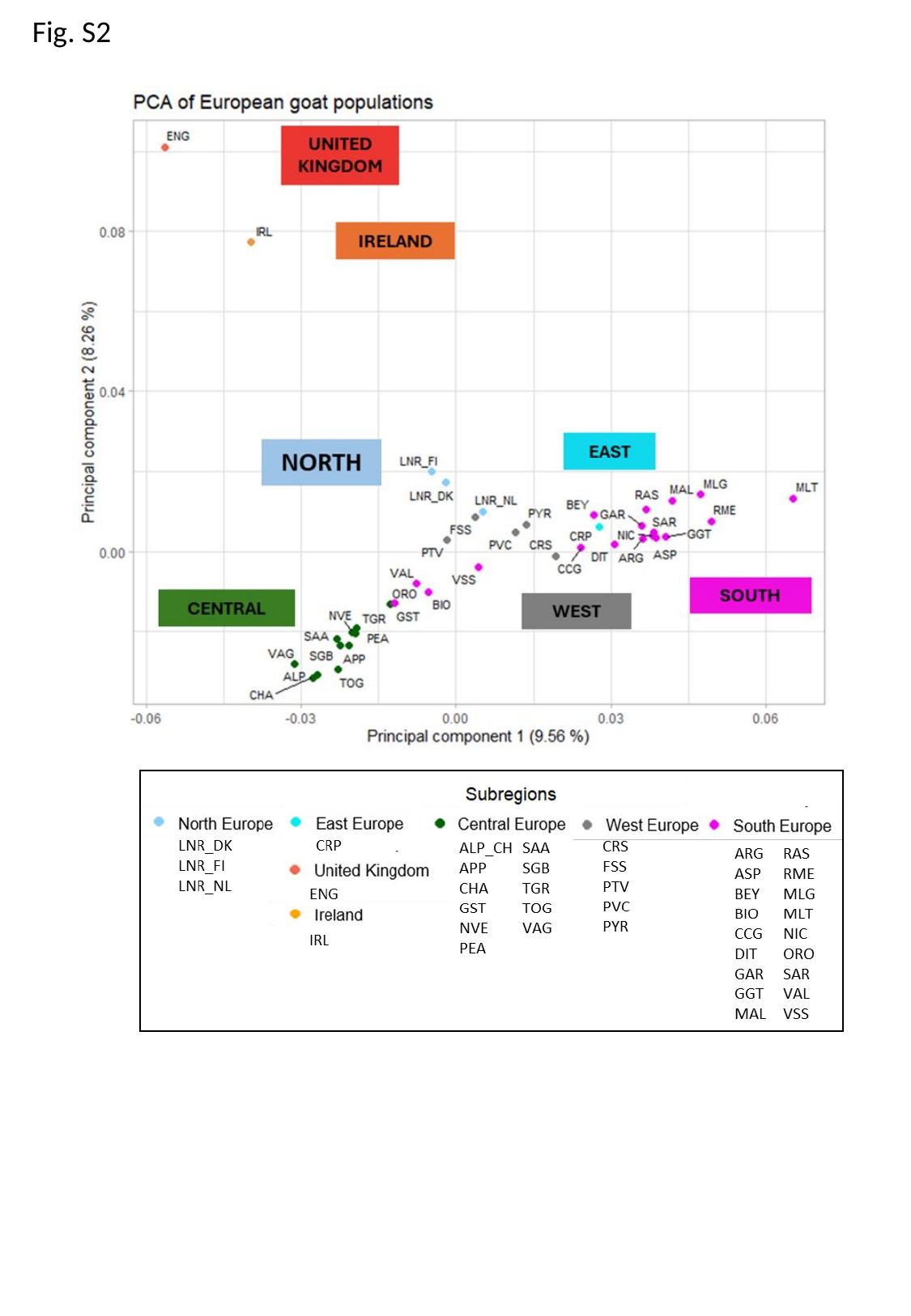

Fig. S2

## Slide 4
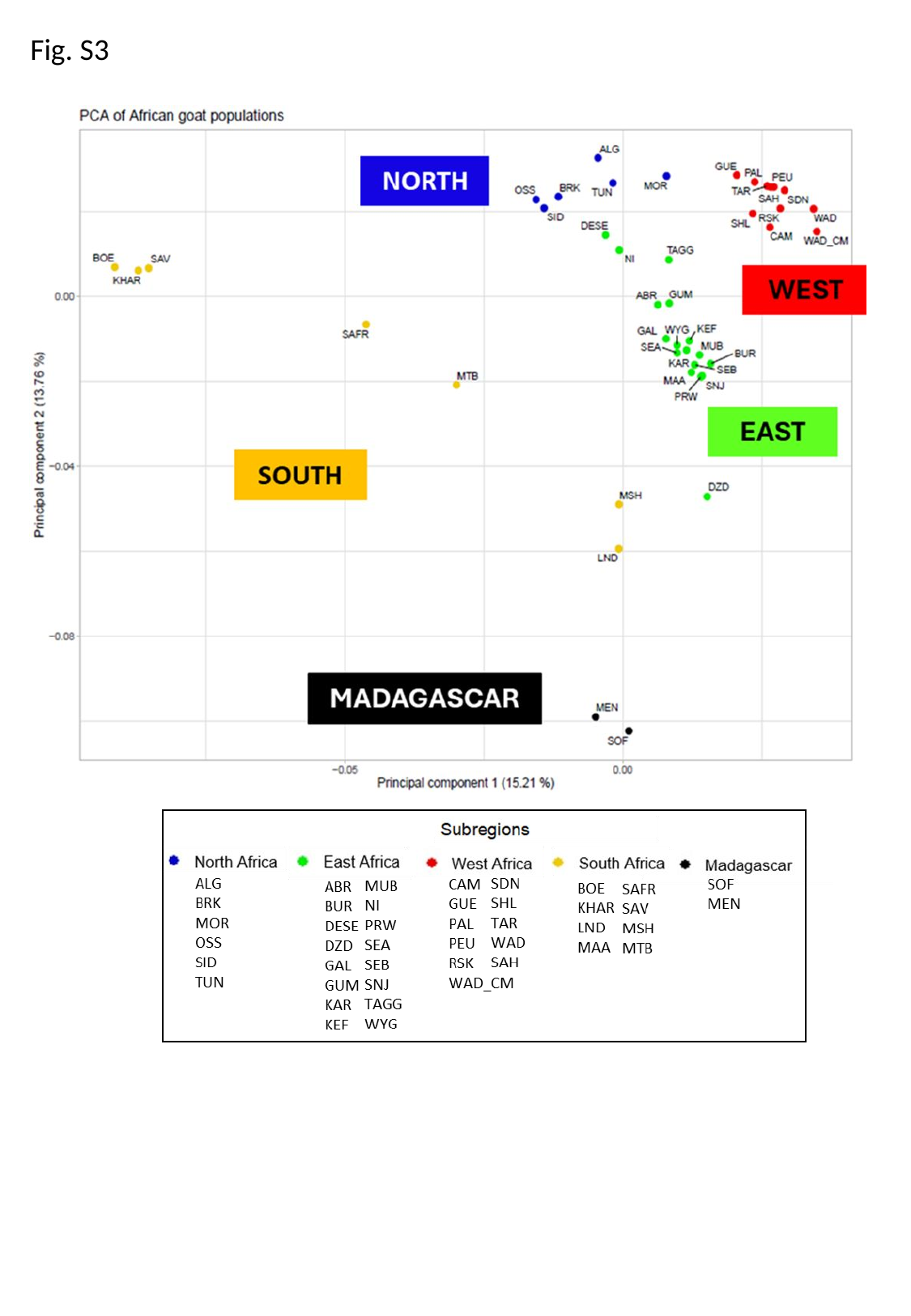

Fig. S3

## Slide 5
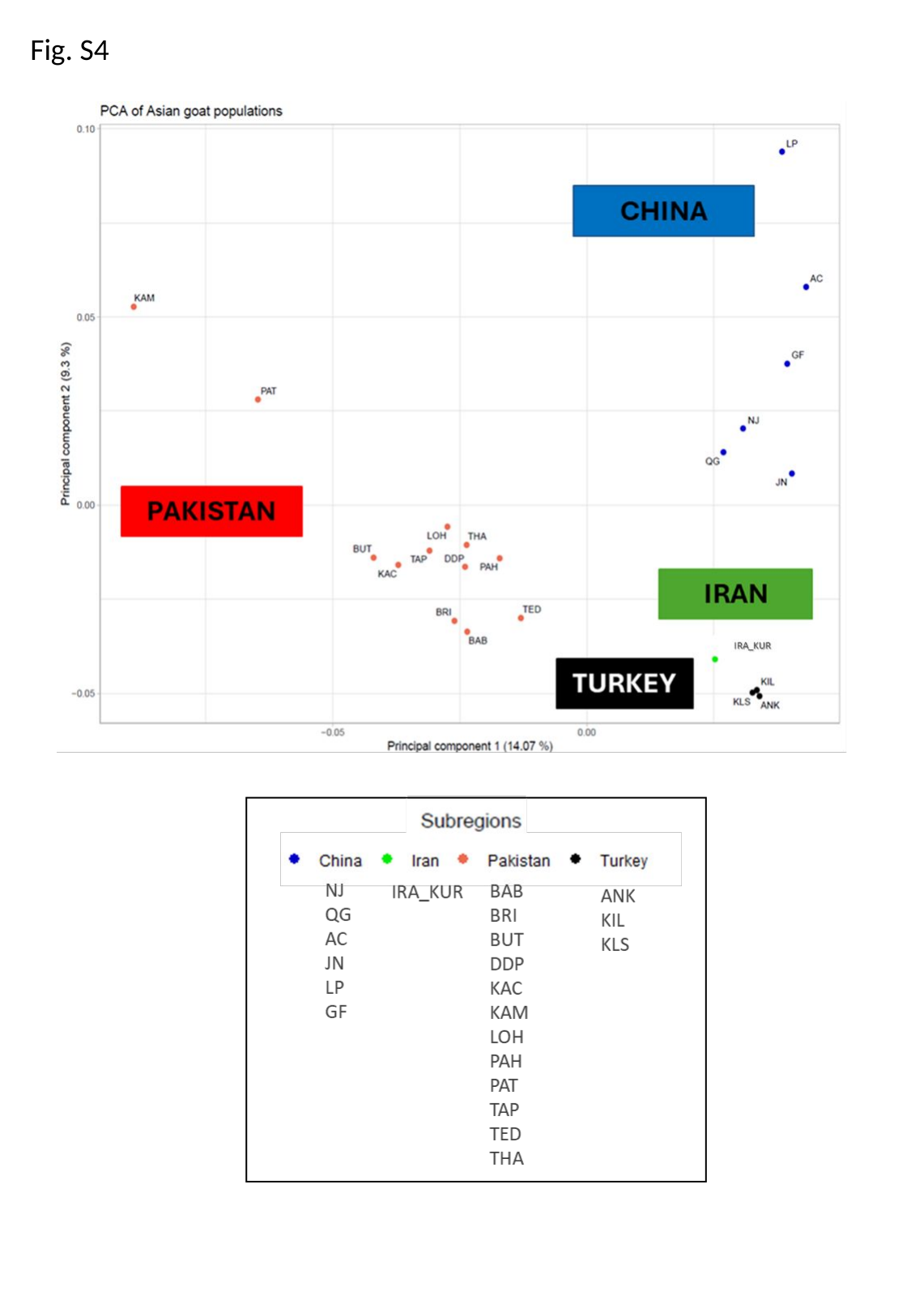

Fig. S4

## Slide 6
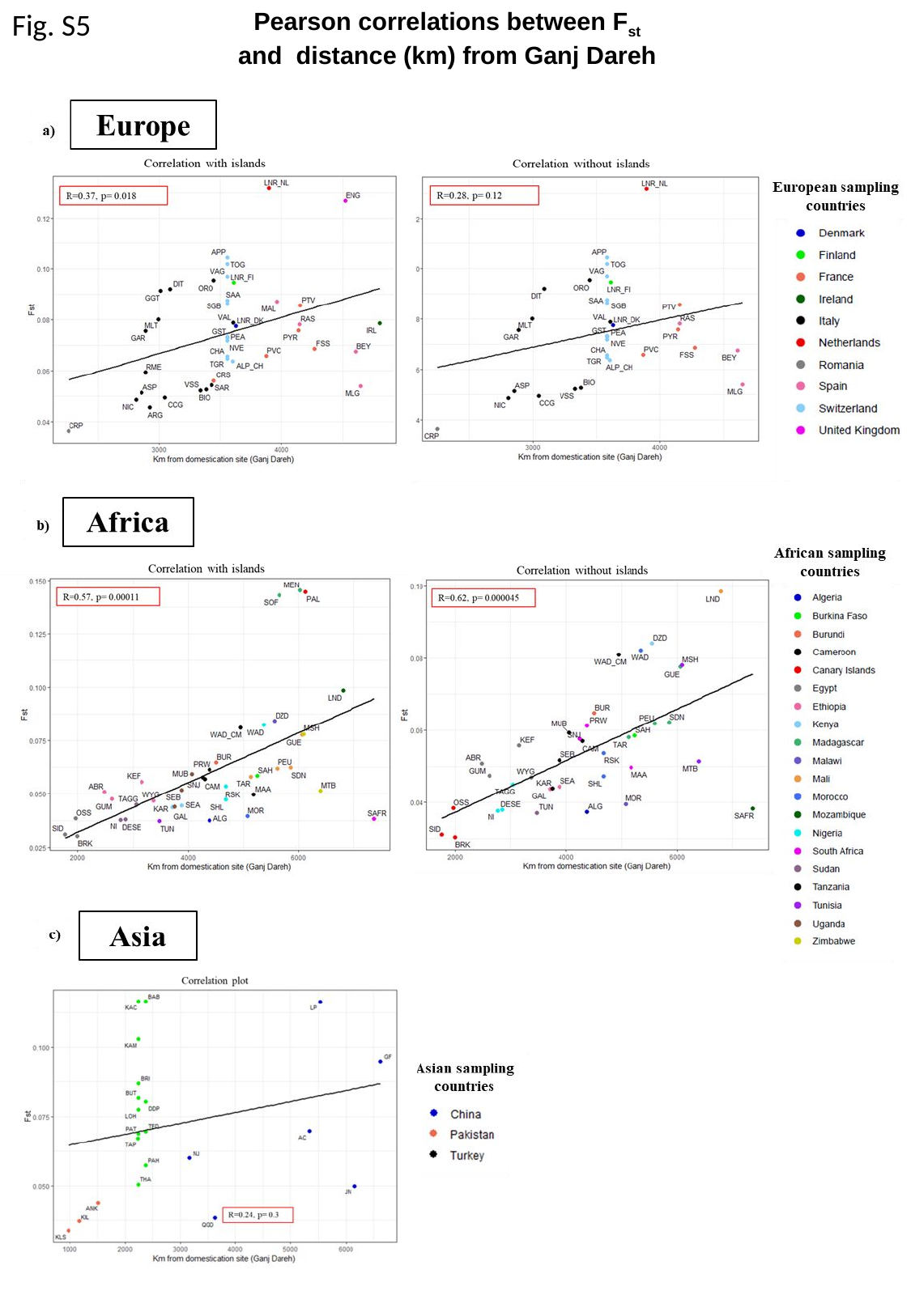

Fig. S5
Pearson correlations between Fst and distance (km) from Ganj Dareh

## Slide 7
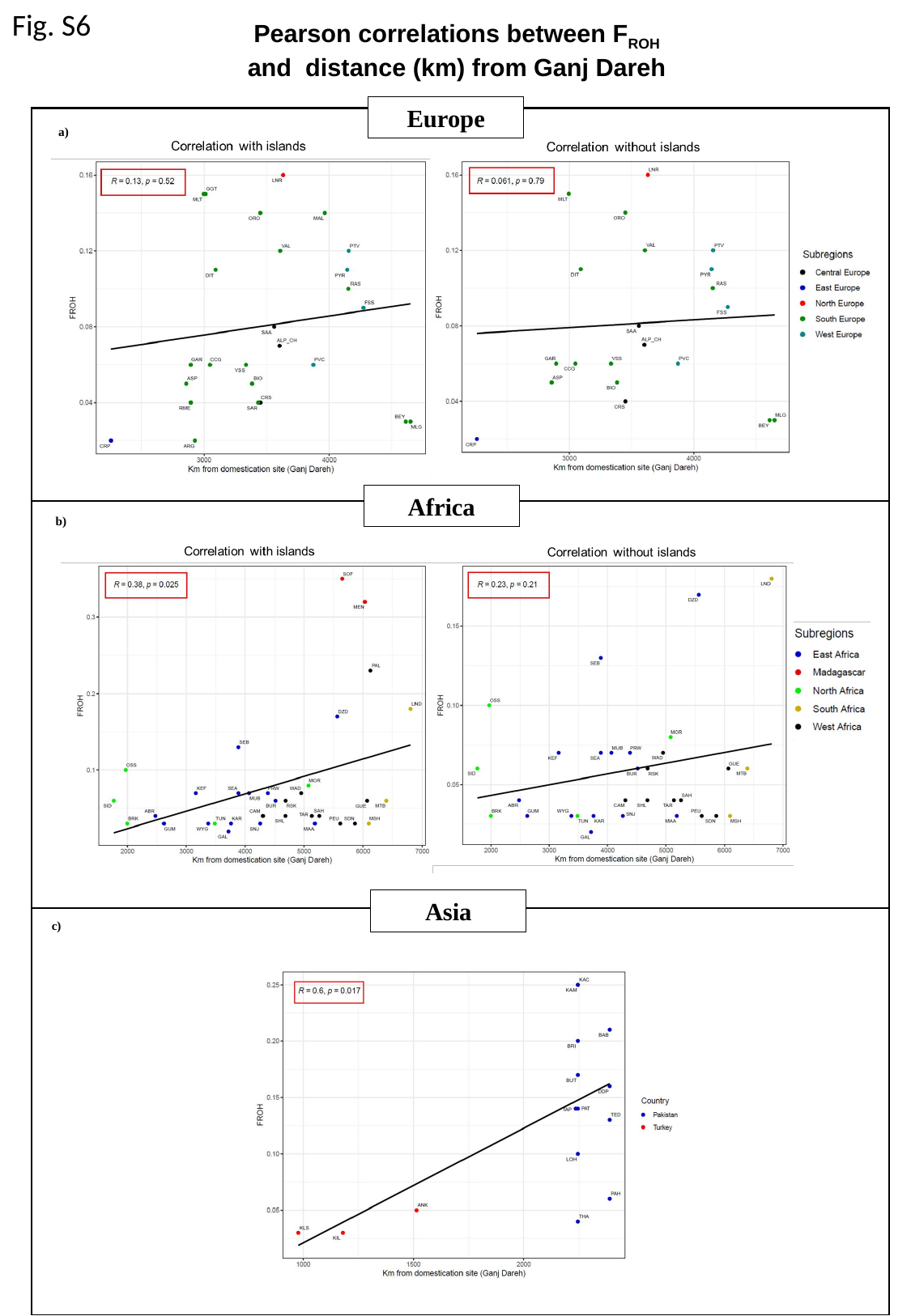

Fig. S6
Pearson correlations between FROH and distance (km) from Ganj Dareh
Europe
a)
Africa
b)
Asia
c)

## Slide 8
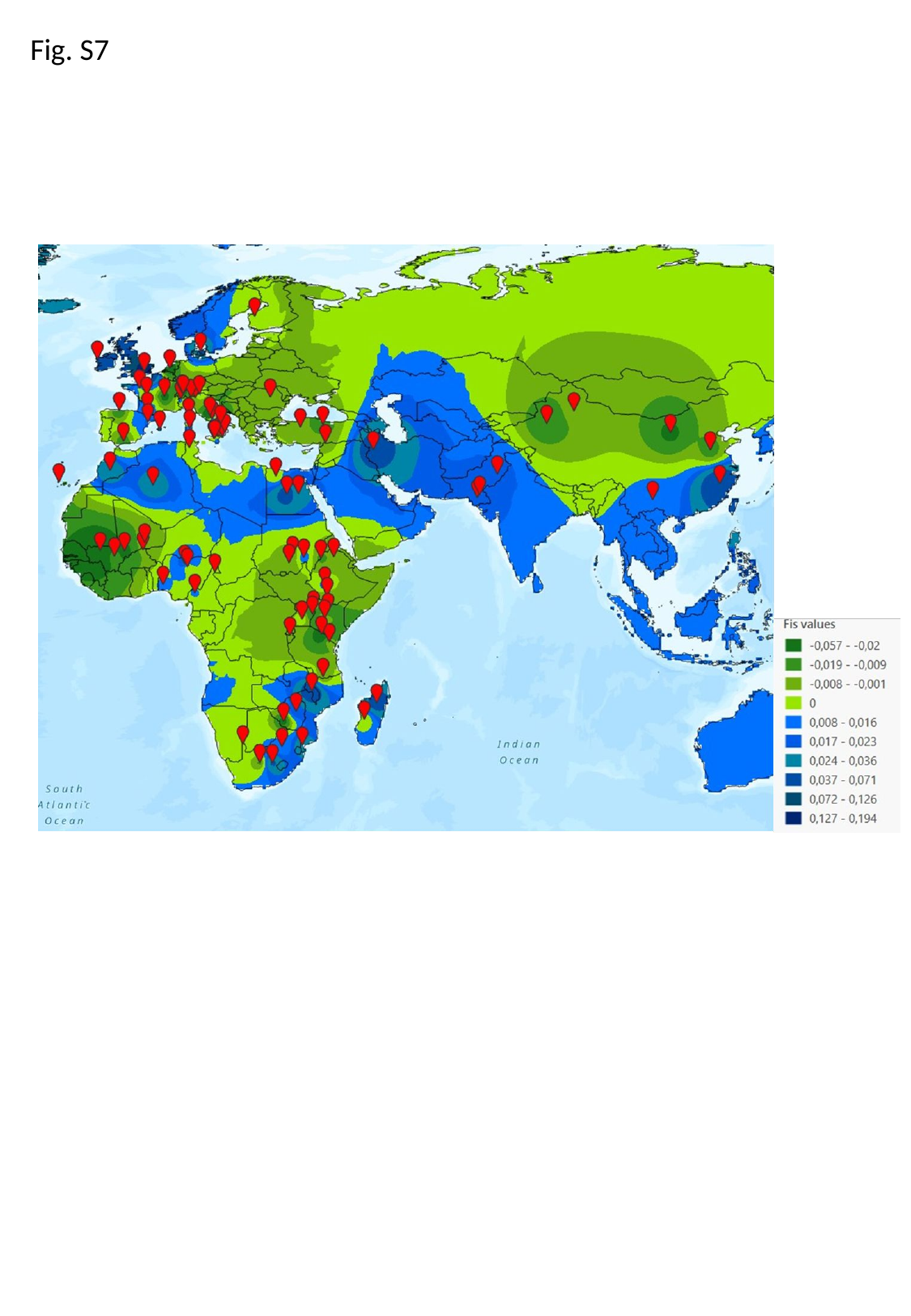

Fig. S7
